# PD-1/SHP-2 negatively regulate Tc1/Th1 phenotypic responses and activation of T cells in the tumor microenvironment

**DOI:** 10.1186/2051-1426-2-S3-P221

**Published:** 2014-11-06

**Authors:** Jing Li, Robert L Ferris

**Affiliations:** 1 Department of Pharmacy, School of Medicine, Tsinghua University, Beijing, China, Pittsburgh, PA, USA; 2 University of Pittsburgh Cancer Institute, Pittsburgh, PA, USA

## 

Rejection of tumor cells by a robust cellular immune response relies on production of type 1 cytokines (such as IFN-γ) and cytolytic activity of T cells. Programmed Death 1 (PD-1), a co-inhibitory receptor proposed to represent T cell dysfunction, is highly expressed on tumor infiltrating lymphocytes (TIL) [1], and may reflect T cell exhaustion marked by decreased proliferation, production of type 1 cytokines and poor cytolytic activity [2]. T-bet, a T-box transcription factor which can be activated by phosphorylated signal transducers and activators of transcription 1 (p-STAT1), plays an important role in Tc1/Th1 skewing. Although anti-PD-1 antibodies enhance IFN-γ secretion after TCR stimulation [3], the mechanistic link between PD-1 and Tc1/Th1 skewing remains unclear. In prospectively collected cancer tissues, TIL manifested dampened Tc1/Th1 skewing and activation compared to PBL (Figure 1 and 2). In addition, PD-1 triggering using PD-L1 coated beads further suppressed TCR-stimulated upregulation of p-STAT1, T-bet and p-S6 as well as Th1 cytokines, while PD-1 blockade reversed suppressive effects of PD-1: PD-L1 ligation (Figure 3). We also found that Src homology-2 domain-containing phosphatase (SHP-2) is higher in TIL than in PBL, tightly correlates with PD-1 expression (Figure 4), and negatively regulates STAT1 and T-bet activation (Figure 5). Thus, the PD-1/SHP-2/p-STAT1/T-bet axis provides an important mechanism for PD-1 suppression of type 1 immunity at tumor sites. PD-1 blocking Abs, which are clinically effective in several solid cancers, should improve T cell-based cancer immunotherapy by restoring robust type 1 immunity and T cell activation to reverse immunosuppression in the tumor microenvironment. SHP-2 inhibitory strategies may also be useful to improve type 1-biased TIL.

**Figure 1:**
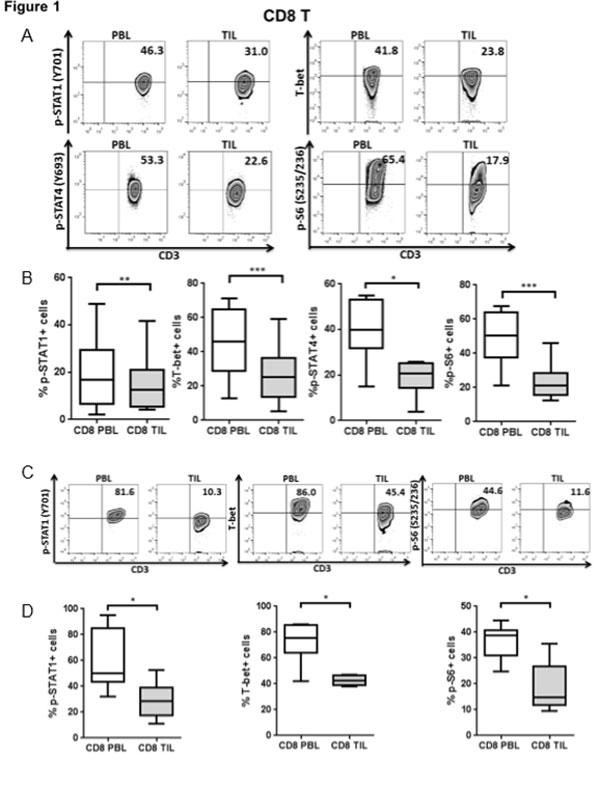
**CD8^+ ^TIL have dampened Tc1 phenotypic responses and activation compared to PBL**. p-STAT1, T-bet, p-STAT4 and p-S6 levels in CD8^+ ^PBL and TIL from HNC patients were analyzed by intracellular flow cytometry. Representative figures (A) and summary data (B) show percentage of p-STAT1 (Y701)+ (n = 15), T-bet+ (n = 16), p-STAT4 (Y693)+ (n = 7) and p-S6 (S235/236)+ (n = 13) cells in CD8^+ ^TIL compared with paired PBL at baseline. Total PBL and TIL were stimulated with anti-CD3/-CD28/hIgG1 beads (bead: cell= 10:1) for 48hrs and then p-STAT1, T-bet and p-S6 were tested by flow cytometry. Representative figures (C) and summary data (D) of percentage of p-STAT1 (Y701)+, T-bet+ and p-S6 (S235/236)+ (n = 6) cells in CD8^+ ^TIL compared with paired PBL post-stimulation are shown. Statistical significance was determined by Wilcoxon (non-parametric paired) test. *p < 0.05, **p < 0.01, ***p < 0.001.

**Figure 2:**
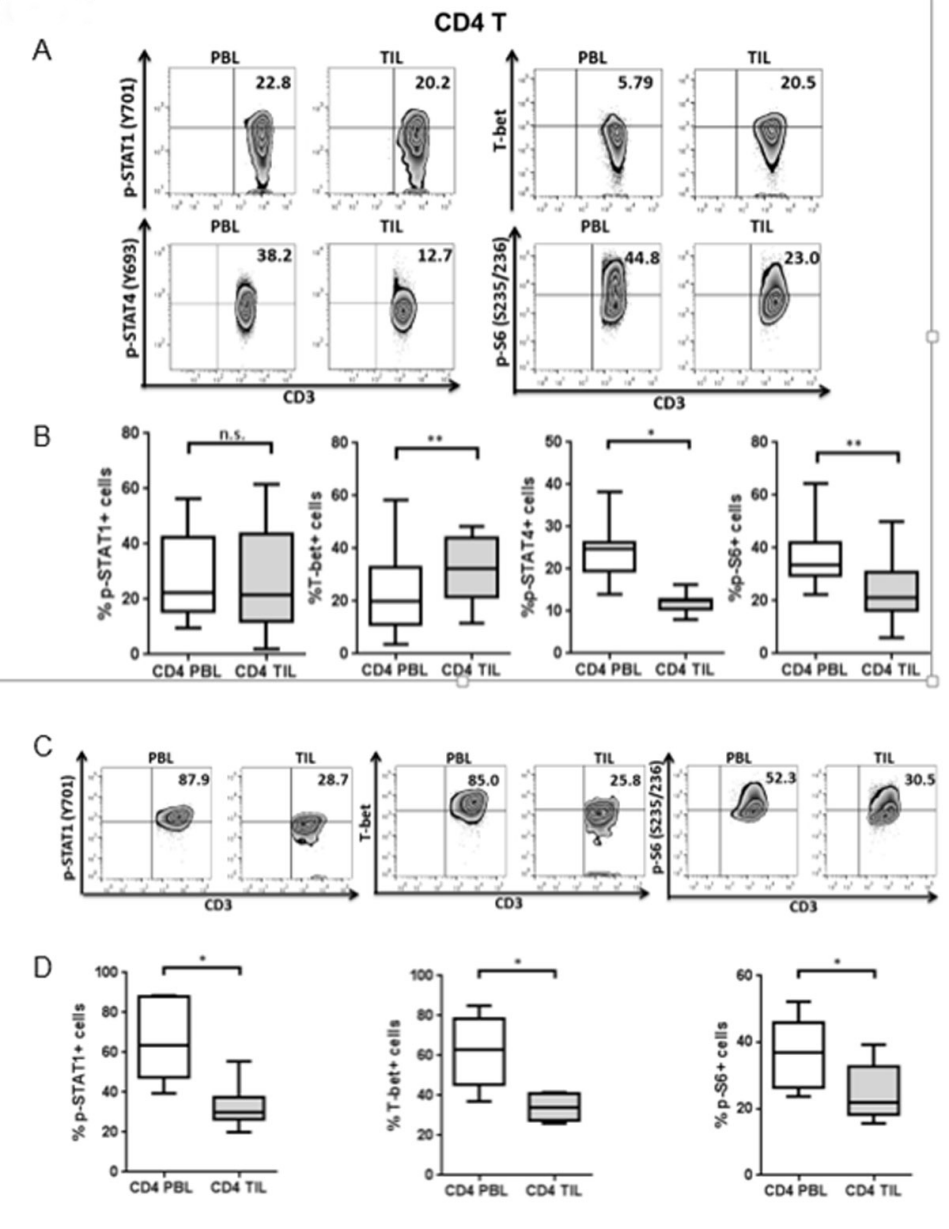
**CD4^+ ^TIL show abortive Th1 differentiation and low activation compared with PBL**. p-STAT1, T-bet, p-STAT4 and p-S6 levels in CD4^+ ^PBL and TIL from HNC patients were analyzed by intracellular flow cytometry. Representative figures (A) and summary data (B) show percentage of p-STAT1 (Y701)+ (n = 15), T-bet+ (n = 16), p-STAT4 (Y693)+ (n = 7) and p-S6 (S235/236)+ (n = 13) cells in CD4^+ ^TIL compared with paired PBL at baseline. Total PBL and TIL were stimulated with anti-CD3/-CD28/hIgG1 beads (bead: cell= 10:1) for 48hrs and then p-STAT1, T-bet and p-S6 were tested by flow cytometry. Representative figures (C) and summary data (D) of percentage of p-STAT1 (Y701)+, T-bet+ and p-S6 (S235/236)+ (n = 6) cells in CD4^+ ^TIL compared with paired PBL post-stimulation are shown. Statistical significance was determined by Wilcoxon (non-parametric paired) test. *p < 0.05, **p < 0.01.

**Figure 3:**
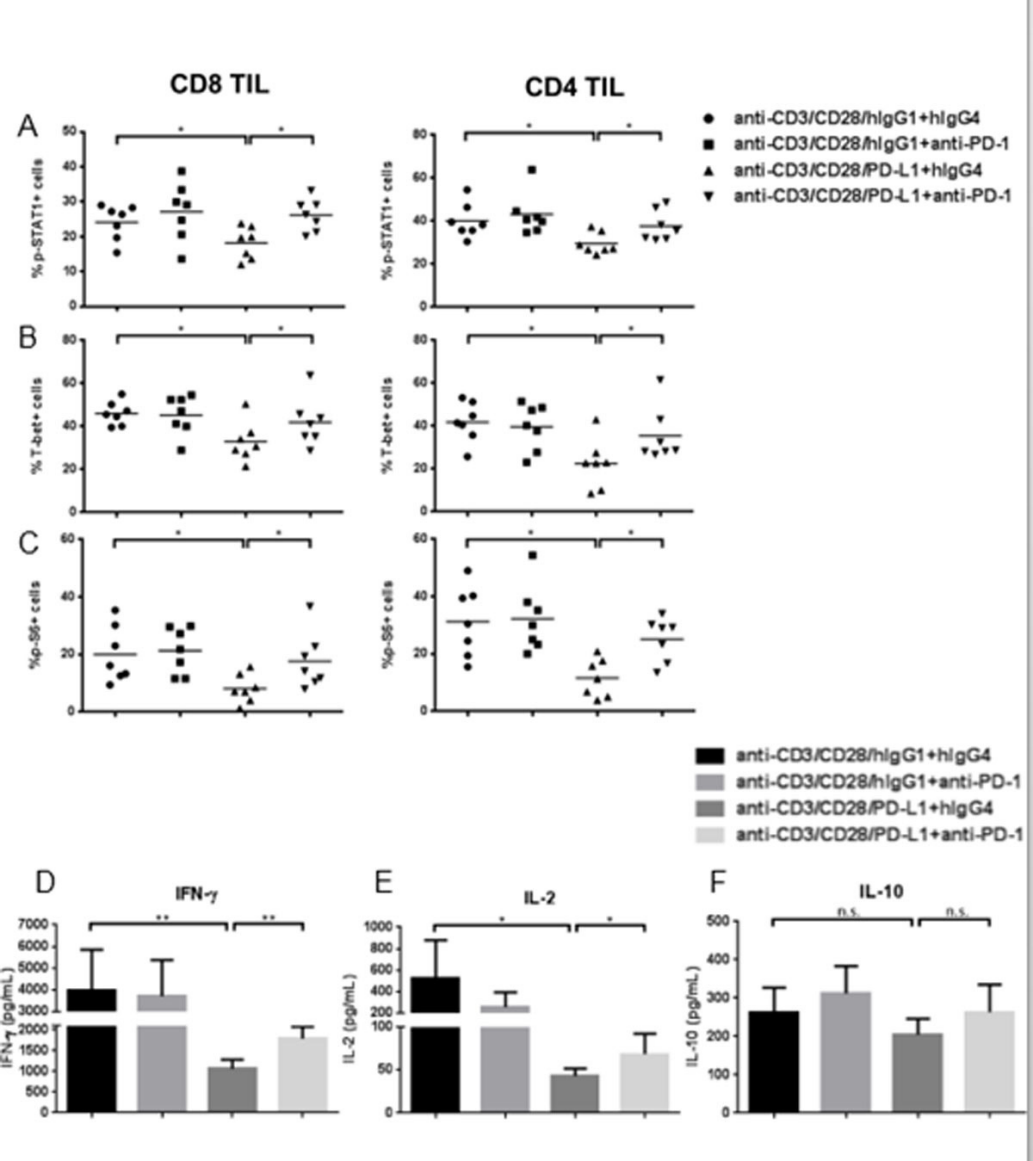
**PD-1 ligation with bead-coated PD-L1 suppressed TCR-stimulated up-regulation of p-STAT1, T-bet and production of Th1 cytokines, while anti-PD-1 blockade could reverse the suppressive effects of PD-1**. Total TIL were stimulated with anti-CD3/-CD28/hIgG1 or anti-CD3/-CD28/PD-L1 coated beads (bead: cell=10:1) for 48h in the presence of 100ug/mL hIgG4 or anti-PD-1 (BMS-936558), then p-STAT1, T-bet and p-S6 were analyzed by flow cytometry. Summary data of frequency of p-STAT1 (Y701)+ (A), T-bet+ (B) and p-S6 (S235/236)+ (C) in CD8+ and CD4+ TIL with indicated conditions is shown (n=7). Supernatants of TIL stimulated with anti-CD3/-CD28/hIgG1 or anti-CD3/-CD28/PD-L1 coated beads (bead: cell=10:1) for 48h in the presence of 100ug/mL hIgG4 or anti-PD-1 (BMS-936558) were collected and stored at -80°. Th1 (IFN-γ and IL-2) and Th2 (IL-10) cytokines in the supernatants were determined by Luminex. Summary data of amount of IFN-Î³ (D), IL-2 (E) and IL-10 (F) in the supernatants of TIL cultured under indicated conditions is shown. The graphs present the mean Â± SEM from 8 HNC patients. Statistical significance was determined by Wilcoxon (non-parametric paired) test. *p<0.05, **p<0.01.

**Figure 4:**
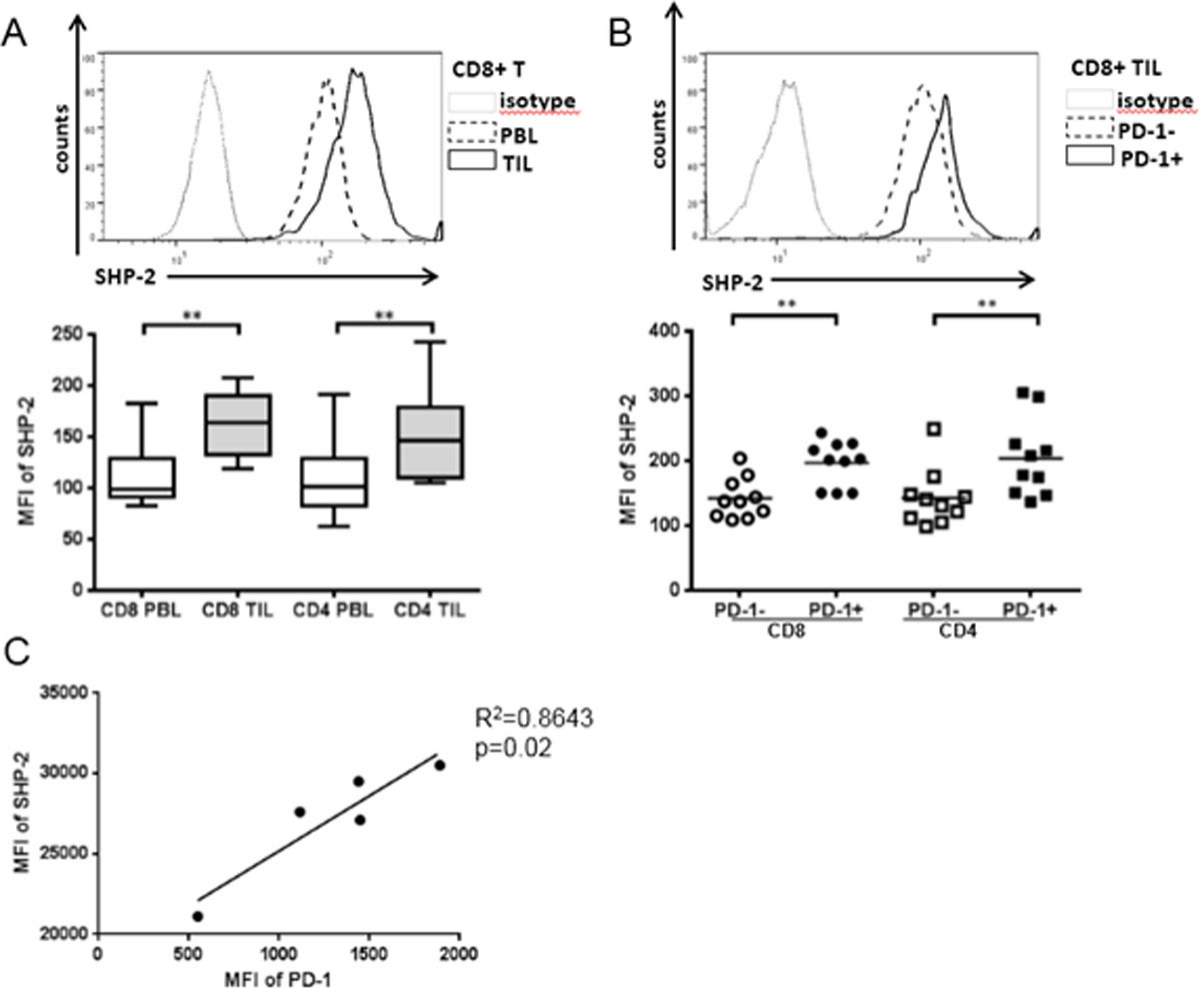
**SHP-2 activation by fusaruside suppresses p-STAT1/T-bet and production of Th1 cytokines upon TCR stimulation**. Total TIL were stimulated with anti-CD3/-CD28/hIgG1 beads (bead: cell = 10:1) or anti-CD3/-CD28/PD-L1 beads plus 100 ug/mL anti-PD-1 blockade (BMS-936558) for 48 h in the presence of 50 uM fusaruside or DMSO. Then p-STAT1 and T-bet were analyzed by flow cytometry. Supernatants were collected and stored at -80°C Th1 (IFN-γand IL-2) and Th2 (IL-10) cytokines in the supernatants were determined by Luminex. A) Summary data of frequency of p-STAT1+ and T-bet+ cells in CD8^+ ^and CD4^+ ^TIL at different conditions is shown (n = 6). B) Summary data of amount of IFN-γ (n = 8), IL-2 (n = 4) and IL-10 (n = 8) in the supernatants of TIL cultured under indicated conditions. The graphs present the mean±SEM from different HNC patients. Statistical significance was determined by Wilcoxon (non-parametric paired) test. *p < 0.05, **p < 0.01.

**Figure 5:**
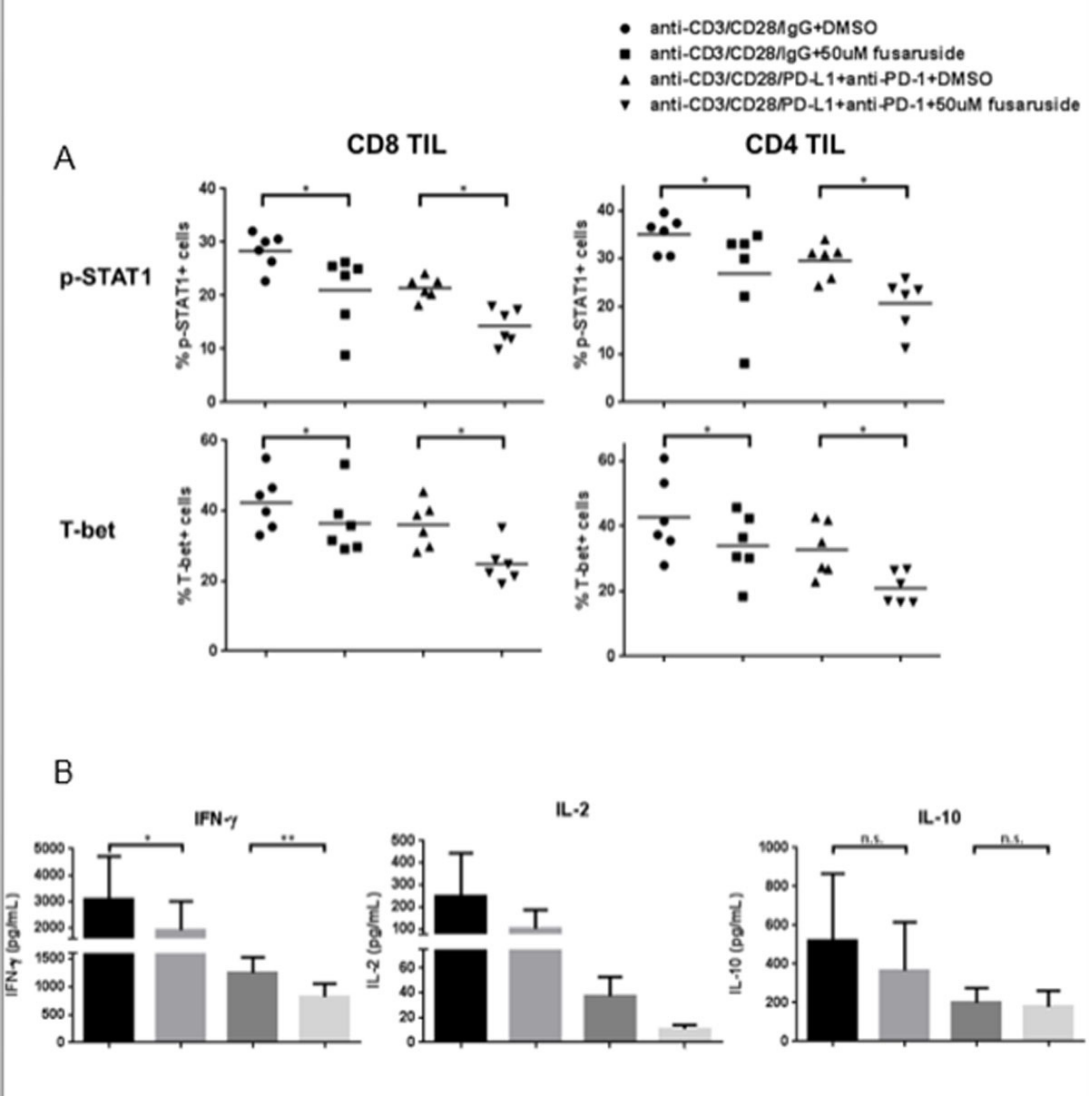
**SHP-2 activation by fusaruside suppresses p-STAT1/T-bet and production of Th1 cytokines upon TCR stimulation**. Total TIL were stimulated with anti-CD3/-CD28/hIgG1 beads (bead: cell = 10:1) or anti-CD3/-CD28/PD-L1 beads plus 100 ug/mL anti-PD-1 blockade (BMS-936558) for 48 h in the presence of 50 uM fusaruside or DMSO. Then p-STAT1 and T-bet were analyzed by flow cytometry. Supernatants were collected and stored at -80. Th1 (IFN-γ and IL-2) and Th2 (IL-10) cytokines in the supernatants were determined by Luminex. A) Summary data of frequency of p-STAT1+ and T-bet+ cells in CD8^+ ^and CD4^+ ^TIL at different conditions is shown (n = 6). B) Summary data of amount of IFN-γ (n = 8), IL-2 (n = 4) and IL-10 (n = 8) in the supernatants of TIL cultured under indicated conditions. The graphs present the mean±SEM from different HNC patients. Statistical significance was determined by Wilcoxon (non-parametric paired) test. *p < 0.05, **p < 0.01.

## References

[B1] Lyford-Pike S Peng S Young GD Evidence for a role of the PD-1:PD-L1 pathway in immune resistance of HPV-associated head and neck squamous cell carcinoma Cancer Res 2013 73 1733 41 10.1158/0008-5472.CAN-12-2384 23288508 PMC3602406

[B2] Wherry EJ T cell exhaustion Nature immunology 2011 12 492 9 21739672 10.1038/ni.2035

[B3] Badoual C Hans S Merillon N PD-1-expressing tumor-infiltrating T cells are a favorable prognostic biomarker in HPV-associated head and neck cancer Cancer Res 2013 73 128 38 23135914 10.1158/0008-5472.CAN-12-2606

